# Development of chloroplast genome resources for peanut (*Arachis hypogaea* L.) and other species of *Arachis*

**DOI:** 10.1038/s41598-017-12026-x

**Published:** 2017-09-14

**Authors:** Dongmei Yin, Yun Wang, Xingguo Zhang, Xingli Ma, Xiaoyan He, Jianhang Zhang

**Affiliations:** College of Agronomy, Henan Agricultural University, Zhengzhou, 450002 China

## Abstract

Peanut (*Arachis hypogaea L*.) is an important oilseed and cash crop worldwide. Wild *Arachis* spp. are potental sources of novel genes for the genetic improvement of cultivated peanut. Understanding the genetic relationships with cultivated peanut is important for the efficient use of wild species in breeding programmes. However, for this genus, only a few genetic resources have been explored so far. In this study, new chloroplast genomic resources have been developed for the genus *Arachis* based on whole chloroplast genomes from seven species that were sequenced using next-generation sequencing technologies. The chloroplast genomes ranged in length from 156,275 to 156,395 bp, and their gene contents, gene orders, and GC contents were similar to those for other Fabaceae species. Comparative analyses among the seven chloroplast genomes revealed 643 variable sites that included 212 singletons and 431 parsimony-informative sites. We also identified 101 SSR loci and 85 indel mutation events. Thirty-seven SSR loci were found to be polymorphic by *in silico* comparative analyses. Eleven highly divergent DNA regions, suitable for phylogenetic and species identification, were detected in the seven chloroplast genomes. A molecular phylogeny based on the complete chloroplast genome sequences provided the best resolution of the seven *Arachis* species.

## Introduction

*Arachis hypogaea* L., known commonly as the peanut or groundnut, is an herbaceous plant belonging to the botanical family Fabaceae. Peanut has a long and relatively complex history which involves natural evolution and human domestication^1^. *A. hypogaea* is an important oilseed and cash crop worldwide, and is mainly cultivated in tropical and subtropical areas as well as in warm parts of temperate regions. China is the largest peanut producer in the world with over 20% of the planting area and more than 40% of the production^2^. *A. hypogaea* is an allotetraploid (AABB-type genome; 2*n* = 4*x* = 40) with a genome size of about 2.7 Gb that is probably derived from a single recent hybridization event between the two diploid species *Arachis ipaensis* (BB genome) and *Arachis duranensis* (AA genome) followed by polyploidization^3^.

The genus *Arachis* is native to South America, and the 80 recognized species have been divided taxonomically into nine sections based on their morphology, geographical distribution, and cross compatibility relationships^4^. Wild *Arachis* spp. offer novel genetic resources for commercial peanut improvement. Thus, knowledge of the genetic relationships within the genus and accurate species identification is important for the efficient use of wild species in breeding programmes for broadening the genetic base of *A. hypogaea* ^1^. For example, the *A. duranensis* genome is a major source of candidate genes for fructification and oil biosynthesis^1^.

Consequently, in the genus *Arachis*, molecular resources have been developed in recent years for genetic characterization, phylogenetics, and domestication studies. A wide range of molecular markers including isozymes and proteins^5,6^, RFLPs^7^, RAPDs^8,9^, AFLPs^10,11^ and microsatellites^2,12–14^ have been used in investgations of genetic diversity and population structure in cultivars/breeding lines and for linkage map construction and QTL analysis. The nrITS (nuclear ribosomal internal transcribed spacer) and 5.8 S rDNA sequences have been used for estimating phylogenetic relationships^15–17^. However, at the genus level, only a few chloroplast genomic resources have been explored. In Genbank, there are presently fewer than 100 sequences from the chloroplast DNA of *Arachis* species.

Chloroplast DNA (cpDNA) is a powerful tool in plant systematics and for the identification of DNA polymorphisms at the inter- and intra-specific levels^18^. The recent availability of next-generation sequencing technologies has enabled the generation of large amounts of DNA sequence data at relatively low cost, which has in turn allowed the exploration of many plant genomes at the molecular level along with novel approaches for phylogenetic studies and breeding strategies. Due to the slower evolution of chloroplast genomes compared to nuclear genomes, chloroplast sequences provide valuable resources that are commonly used in studies of population genetics, phylogeny, phylogeography, and species identification^19–21^.

Chloroplasts are key photosynthetic organelles in plants that provide energy to green plants. The chloroplast genome is a highly conserved circular DNA molecule ranging in size from 115 to 165 kb. The cpDNA genome usually carries two copies of a large inverted repeat (IR) separated by small (SSC) and large (LSC) single-copy regions. Approximately 130 genes are encoded by the circular chloroplast genome, which exhibits a highly conserved gene order and content, and typically encodes 79 proteins, 30 transfer RNAs, and four ribosomal RNAs^22,23^. The number of sequenced plant plastid genomes increased rapidly during the last decade due to the implementation of next-generation DNA sequencing technologies^24^. The number of chloroplast genomes from land plants released by the National Center for Biotechnology Information (NCBI) has risen to 1540 (accessed March 7, 2017). Complete chloroplast genome sequences are widely accepted as informative and valuable data sources for studies in evolutionary biology.

In this study, we sequenced the chloroplast genomes of seven *Arachis* species using a next-generation sequencing platform. Our aim was to retrieve valuable chloroplast genome information, such as SNPs, microsatellites, indels, and highly variable regions for this genus, by comparing the chloroplast genomes to one another. Our second objective was to assess phylogenetic relationships among the seven *Arachis* species. Our results will provide abundant molecular tools for further species identification, phylogenetic resolution, and population genetics, and will also assist in breeding in *Arachis* species.

## Results

### Chloroplast genome sequencing, assembly, and validation

Using the Illumina HiSeq. 4000 system, total DNA from seven species of *Arachis* was sequenced to produce 11,732,639–17,815,336 paired-end raw reads (150 bp average read length) per species. All sequences were assembled by first using a de novo assembly and then a reference-based assembly. To validate the accuracy of the assembled chloroplast genome, four junction regions and all gaps between all contigs were validated by Sanger-based sequencing in each of the seven chloroplast genomes. The finished, high quality chloroplast genome sequences thus obtained were used in the following analyses and were submitted to GenBank.

### Chloroplast genome structural features and gene content

The assembled genomes of all seven *Arachis* species are collinear with previously published chloroplast genomes of *Arachis* ^25^, because no rearrangements were identified. The *Arachis* chloroplast genomes ranged from 156,275 to 156,395 base pairs in length, with *A. hypogaea* being the largest and *A. batizocoi* the smallest. All of the *Arachis* chloroplast genomes displayed the typical quadripartite structure of angiosperm cpDNA, which consists of a pair of IR regions (25,813–25,824 bp) separated by a LSC region (85,863–85,951 bp), and a SSC region (17,786–17,849 bp). The guanine-cytosine (GC) contents of the cpDNA for the seven species were very similar, around 36.4% (Table 2).

**Figure 1:**
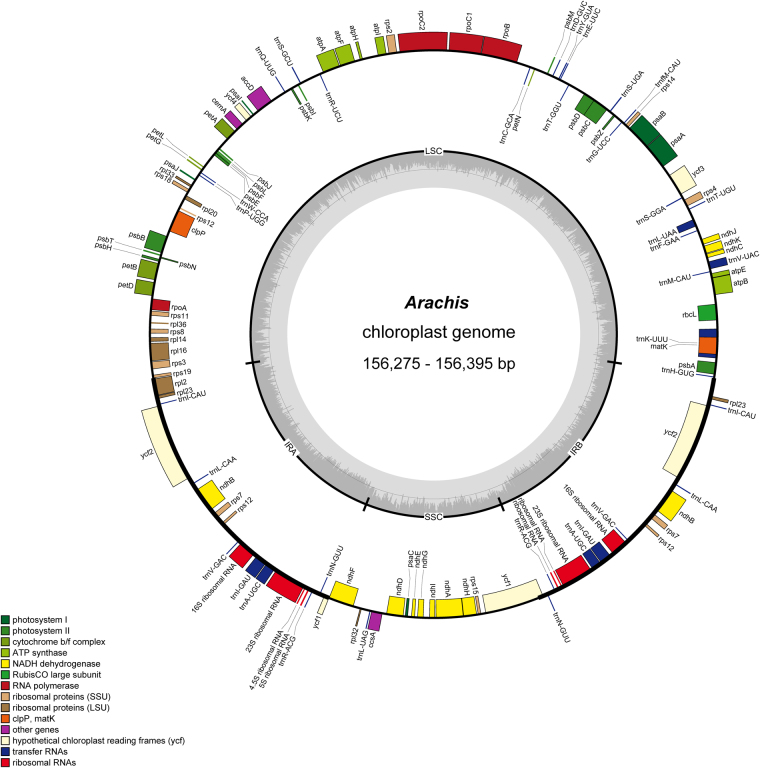
Map of the *Arachis* chloroplast genome. The genes inside and outside of the circle are transcribed in the clockwise and counterclockwise directions, respectively. Genes belonging to different functional groups are shown in different colors. Thick lines indicate the extent of the inverted repeats (IRa and IRb) that separate the genomes into small single-copy (SSC) and large single-copy (LSC) regions.

When the duplicated genes in the IR regions were counted only once, the seven *Arachis* chloroplast genomes all have 110 different genes arranged in the same order, including 76 protein-coding genes, 30 tRNAs, and 4 rRNAs. Seven genes that include one tRNA gene, four rRNA genes, and five protein-coding genes (*rpl23*, *ycf2*, *ndhB*, *rps7*, *ycf15*) are completely duplicated in the IR regions. Twelve of the protein-coding genes and six of the tRNA genes contain introns; 15 of these contain a single intron, whereas three genes have two introns (Fig. 1, Table 1). To detect a possible IR expansion, the IR-LSC/SSC borders with full annotations for the adjacent genes were compared across the seven chloroplast genomes analysed, but no differences were found.

**Figure 2:**
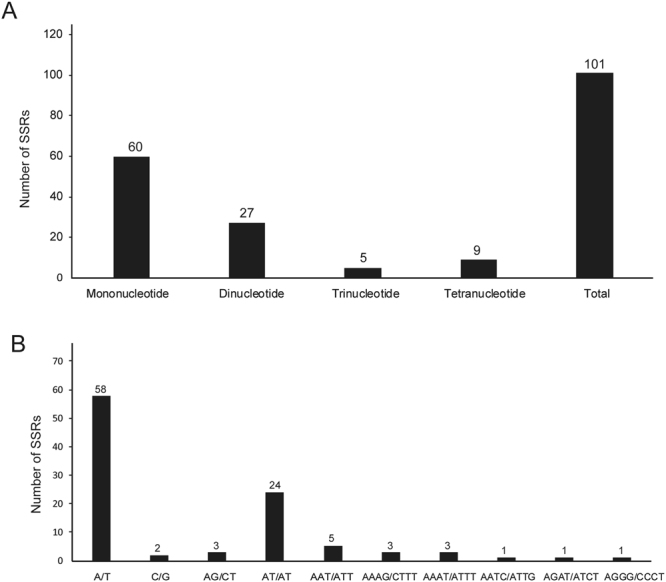
Analyses of simple sequence repeat (SSR) in the *Arachis* chloroplast genomes. **(A)** Number different SSRs types detected by MISA. **(B)** Frequency of identified SSR motifs in the different repeat classes.

**Table 1:** Genes identified in the chloroplast genome of *Arachis* species.

Category for genes	Group of gene	Name of gene
Photosynthesis related genes	Photosystem I	*psaA, psaB, psaC, psaI, psaJ*
	Photosystem II	*psbA, psbB, psbC, psbD, psbE, psbF, psbH, psbI, psbJ, psbK, psbL, psbN, psbT, psbZ*
	cytochrome b/f compelx	petA, *petB, *petD, petG, petL, petN
	ATP synthase	atpA, atpB, atpE, *atpF, atpH, atpI
	cytochrome c synthesis	*ccsA*
	Assembly/stability of photosystem I	*ycf3,ycf4
	NADPH dehydrogenase	*ndhA, *ndhB, ndhC, ndhD, ndhE, ndhF,ndhG, ndhH, ndhI, ndhJ, ndhK
	Rubisco	*rbcL*
Transcription and translation related genes	transcription	rpoA, rpoB, *rpoC1, rpoC2
	ribosomal proteins	rps2, rps3, rps4, rps7, rps8, rps11, *rps12, rps14,rps15, *rps16, rps18, rps19, rpl2, rpl14, *rpl16, rpl20, rpl22, rpl23, rpl32, rpl33,rpl36
RNA genes	ribosomal RNA	*rrn5, rrn4.5, rrn16, rrn23*
	transfer RNA	*trnA-UGC, trnC-GCA, trnD-GUC, trnE-UUC, trnF-GAA,trnG-GCC, *trnG-UCC, trnH-GUG, trnI-CAU, *trnI-GAU,*trnK-UUU, trnL-CAA, *trnL-UAA, trnL-UAG, trnfM-CAU,trnM-CAU, trnN-GUU, trnP-UGG, trnQ-UUG,trnR-ACG, trnR-UCU, trnS-GCU, trnS-GGA, trnS-UGA, trnT-GGU,trnT-UGU, trnV-GAC, *trnV-UAC, trnW-CCA, trnY-GUA
Other genes	RNA processing	*matK*
	carbon metabolism	*cemA*
	fatty acid synthesis	*accD*
	proteolysis	*clpP
Genes of unknown function	conserved reading frames	*ycf1, ycf2*

Intron-containing genes are marked by asterisks (*).

**Table 2:** Details of the complete chloroplast genomes of seven *Arachis* species.

	*A. appressipila*	*A. batizocoi*	*A. diogoi*	*A. helodes*	*A. hypogaea*	*A. rigonii*	*A. villosa*
Total	156,394	156,275	156,393	156,378	156,395	156,343	156,381
LSC	85,946	85,863	85,951	85,934	85,951	85,868	85,932
SSC	18,800	18,786	18,794	18,796	18,796	18,849	18,801
IR	25,824	25,813	25824	25,824	25,824	25,813	25,824
Total	110	110	110	110	110	110	110
Protein coding genes	76	76	76	76	76	76	76
rRNA	4	4	4	4	4	4	4
tRNA	30	30	30	30	30	30	30
GC%	36.4%	36.4%	36.4%	36.4%	36.4%	36.4%	36.4%

### Chloroplast genome sequence divergence among *Arachis* species

The seven chloroplast genomes were fully aligned, giving an alignment matrix of 156,818 bp. The alignment revealed a high degree of sequence similarity across the *Arachis* chloroplast genome, which suggests that it is highly conserved in *Arachis*. We retrieved 643 variable sites (0.41%), including 212 singletons and 431 parsimony-informative sites (0.27%) across the entire chloroplast genome (Table 3).

**Table 3:** Variable site analyses in the seven *Arachis* chloroplast genomes.

		Variable sites		Information sites		Nucleotide Diversity
	Number of sites	Numbers	%	Numbers	%	
LSC	88,262	460	0.52%	298	0.34%	0.00185
SSC	18,898	135	0.71%	91	0.48%	0.0025
IR	25,829	24	0.09%	21	0.08%	0.00037
Complete cp genome	156,818	643	0.41%	431	0.27%	0.00144

**Figure 3:**
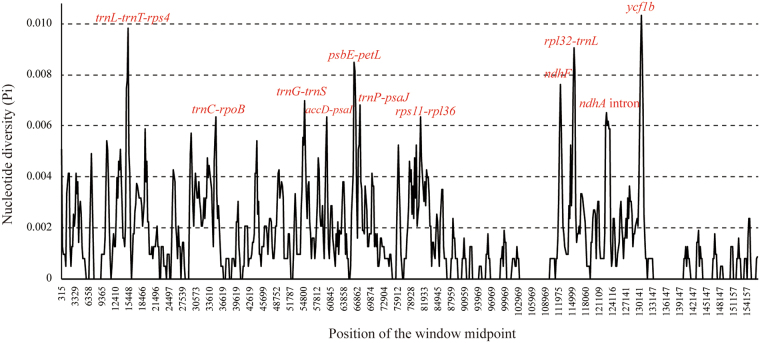
Sliding window analysis of the complete chloroplast genomes of seven *Arachis* species (window length: 600 bp, step size: 200 bp). X-axis: position of the window midpoint, Y-axis: nucleotide diversity within each window.

To elucidate the level of sequence divergence, the nucleotide variability (π) values within 600 bp windows in the seven *Arachis* chloroplast genomes were calculated with DnaSP 5.0 software. The variability throughout the chloroplast genomes was quantified using the average nucleotide diversity (π) (Fig. 3). The average value of π is 0.00166. Among the LSC, SSC, and IR regions, the SSC exhibits the highest nucleotide diversity (0.0025), and the IR exhibit the least divergence (0.00037). There were eleven peaks which showed remarkably higher π values (>0.006). Two are in the coding regions of *ndhF* and *ycf1*, one is in the *ndhA* intron, and nine are in the intergenic regions (*accD-psaI*, *psbE-petL*, *rps11-rpl36*, *rpl32-trnL*, *trnC-rpoB*, *trnG-trnS*, *trnL-trnT-rps4*, *trnP-psaJ*). Seven of these regions lie in the LSC and four are in the SSC.

The number of nucleotide substitutions and p-distances and were used to estimate the divergence between the seven *Arachis* species. The number of nucleotide substitutions in pairwise comparisons between the seven species ranged from 23 to 433, and the p-distances ranged from 0.00015 to 0.00278 (Table 4). The overall sequence divergence estimated by p-distance among the four genomes was only 0.00167. The cp genomes of *A. batizocoi* and *A. rigonii* had the lowest levels of sequence divergence.

**Table 4:** Nucleotide substitutions and sequence divergence in seven complete chloroplast genomes in *Arachis*.

	*A. appressipila*	*A. batizocoi*	*A. diogoi*	*A. helodes*	*A. hypogaea*	*A. rigonii*	*A. villosa*
*A. appressipila*		0.00272	0.00086	0.00095	0.00092	0.00271	0.00088
*A. batizocoi*	424		0.00270	0.00281	0.00275	0.00015	0.00273
*A. diogoi*	134	421		0.00057	0.00061	0.00266	0.00074
*A. helodes*	149	438	89		0.00058	0.00278	0.00081
*A. hypogaea*	144	428	96	91		0.00274	0.00077
*A. rigonii*	422	23	415	433	427		0.00270
*A. villosa*	138	425	116	126	121	420	

The lower triangle shows the number of nucleotide substitutions between the genomes. The upper triangle indicates the calculated sequence divergence for the seven complete chloroplast genomes.

### Indels and microsatellites

**Figure 4:**
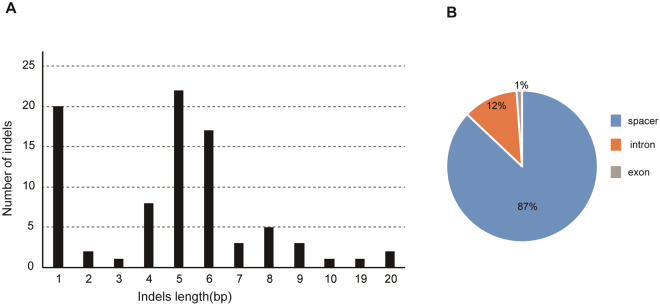
Indels identified in the cp genomes of seven *Arachis* species. (**A**) Numbers of individual indels shown by sequence length. (**B**) Relative frequency of indel occurrence in introns, exons, and spacer regions.

The indels were filtered to separate common indel events from all SSR-indel mutations in this study. We adopted a simple and straightforward strategy for identifying common indel mutations. We retrieved 85 common indels from the cp genomes of all seven *Arachis* species (Table S2). Only one was found in a genic region (*ycf1*). Ten (12%) were located in intronic regions, including *atpF*, *clpP*, *ndhA* (two indels), *petB*, *petD*, *trnG*, *trnV* and *ycf3* (two indels). Forty-four spacer regions harboured indels; the *psbD-trnT* spacer had the highest number of indels (seven), followed by *atpH-atpF* (five), *pebM-petN* (four), *trnV-ndhC* (three), and *rpl32-trnL* (three). The sizes of the common indels ranged from 1 to 20 bp, with indels of 1, 4, and 6 bp being the most common (Fig. 4). The largest one, in *ndhF-rpl32*, was a deletion in the *A. batizocoi* cp genome, while the next largest, which was found in *ndhE-ndhG*, was an insertion in *A. rigonii*. Most common indels found in the chloroplast genomes provided phylogenetic signal at the species level.

With MISA analysis, 101 universal SSR loci were detected in the chloroplast genomes of all seven *Arachis* species. Among the SSRs identified, we found 60 mononucleotide motifs that ranged in length from 10 to 15 nucleotides, 27 dinucleotide, 5 trinucleotide, and 9 tetranucleotide SSRs. The majority of the SSRs consist of A/T mononucleotide repeats. Chloroplast genome SSRs are composed of A and/or T and rarely contain tandem C and/or G repeats. Thirty-seven SSR loci showed polymorphism after *in silico* comparative analysis (Fig. 2, Table S3). Intergenic regions contained 30 SSRs, and there were seven located in intronic regions. We designed primer pairs for amplification of all the SSRs (Table 5, Table S4).

**Table 5:** SSRs identified from *in silico* comparative analysis of the seven *Arachis* cp genomes.

No.	Position	Region	Locatin	SSR type	Forward sequence	Reverse sequence	Length (bp)
1	trnK-rbcL	LSC	spacer	(A)10	TACCATTGAGTTAGCAACCCCC	CGATTTCTTCACGTTACAGAGGC	248
2	trnK-rbcL	LSC	spacer	(A)12	CGATTTCTTCACGATCGGATTA	AATATAATCAAATTCGATTTA	141
3	rbcL-atpB	LSC	spacer	(A)12	TCATATGTATGGCGCAACCCAA	TTCATGGGCGAGCATACAATTT	189
4	trnV intron	LSC	intron	(T)12	TCAAAAACGCAAGGGCTATAGC	TACTGGACGTCTCAACCCTTTG	190
5	trnF-trnL	LSC	spacer	(A)15	ACTCGAATCCATTTGTGAAAGACT	TCCCTCTATCCCCAAAAGACCT	131
6	trnL-trnT	LSC	spacer	(T)10	TTGCGATTAGAATCGCATTAA	AGATTCGACAAAATCTGGATA	151
7	trnL-trnT	LSC	spacer	(T)11	ATTACTGTAACTGTAATAGAA	ATGCTCTAACCTCTGAGCTA	246
8	ycf3 2nd intron	LSC	intron	(A)11	TGATCTGTCATTACGTGCGACT	TCTTTACGGCGCTTCCTCTATC	208
9	ycf3-psaA	LSC	spacer	(T)12	TGAAGATCACAGGGCGTTCTTA	TGGATGGACTGATGTAGACAACA	280
10	ycf3-psaA	LSC	spacer	(AT)7	TAGTTCTATTTATATTATTC	ATTTAAATGAAATATGCATTA	143
11	ycf3-psaA	LSC	spacer	(T)10	ATTCAAAAAGGTCCGTTGAGCG	CTCCTTCCGGACAACACATACA	230
12	psbD-trnT	LSC	spacer	(A)14	GTGAAGCCATGATTTGATGTA	ATTAGTCGATATTTACGATTA	193
13	psbD-trnT	LSC	spacer	(A)10	GAATCTTGAGGAACGGGAGGAT	AGTGGACCTAACCCATTGAATCA	158
14	psbD-trnT	LSC	spacer	(T)13	TTGATTATCATTCATTAGAAT	GTAAGGCGTAAGTCATCGGT	243
15	trnT-trnE	LSC	spacer	(A)12	TCCTGCTCTTGAACCGATTCTT	GTTGGTTTGCTAGAAAAGGCGT	188
16	trnT-trnE	LSC	spacer	(G)11	TGGAATTATAGATTGGCGATT	ATGTCCTGGACCACTAGACGA	223
17	trnD-psbM	LSC	spacer	(A)13	CCCGTCAGTCCCGAATGAATAA	CGATTCATCGTCGAGAATGGAA	256
18	petN-trnC	LSC	spacer	(T)10	AAGATTTACTATATCCATGTG	TTGACTCTGTACCAGCGATT	182
19	trnC-rpoB	LSC	spacer	(AT)6	GAAAAAGGATTTGCAGTCCCCC	GGTTCCGTTTTGTCCTTCCATT	140
20	trnC-rpoB	LSC	spacer	(A)10	GGTGTGTAAACTCTCCCACCTT	AAATCGACTCGGGATTTGTTCG	227
21	atpH-atpF	LSC	spacer	(T)10	TACAAGCGGTATTCAAGCCCT	CAATTAATAGAATCAGAATTCA	227
22	atpH-atpF	LSC	spacer	(T)11	ATTCAGTTCTTCGGTCGAACGA	ACCGTAAACCAATTGTTCGTGT	259
23	atpF-intron	LSC	intron	(A)10	AAAGCAAAGCTAGGCATAGGCA	ACGTAGGTCATCGATTTCGCAT	259
24	trnQ-accD	LSC	spacer	(a)13	TGCAAGCAAAAGTGTATTCCGG	ACTTGGTCCAGGATCTTTTAGCT	167
25	psaJ-rpl33	LSC	spacer	(T)10	CTATTGATCGAAATCAATCGT	CCATTGAAGCCTGTACCAGAT	235
26	rpl20-rps12	LSC	spacer	(T)12	GAGTTGGTTTAGATCAATCT	ATGTCAGCAGCAGAAGCTCA	231
27	rps12-clpP	LSC	spacer	(A)14	GTGACATTTCGGATTGGCTGTC	ATTGTTGATCTTGTCGCGGTTG	276
28	clpP intron 1	LSC	intron	(T)15	AGATCAGCATCAGTAAATGAT	ATCGGAAGCCTATTTCAGTGTC	249
29	clpP-psbB	LSC	spacer	(A)11	CACACCACCATTGCGTATTGTT	GAACACGATACCAAGGCAAACC	271
30	rps11-rpl36	LSC	spacer	(TA)6	GAGATGTATGGATATATTCAT	TTGAATGAATATAGAAATTCTA	297
31	rps11-rpl36	LSC	spacer	(T)11	AGTTTGAATTTCAATATCTA	GATCCGAGATTAAGTTGAAGGA	251
32	rpl16 intron	LSC	intron	(TA)7	TCTACAATGGAGCCTCGCAAAT	ACAAATCAAGAGCACCGAGTCA	104
33	rpl16 intron	LSC	intron	(TTTC)4	TGTTGATGCTTTATTACACTTCCCC	TCATCGCTTCGCATTATCTGGA	272
34	rpl2 intron	IR	intron	(T)10	TTGCAATCAGTTTCGCTACAGC	CTTGTACAGTTTGGGAAGGGGT	161
35	ndhF-rpl32	SSC	spacer	(A)10	GAACTGGAAGCGGAATGAAAGG	AGAAGTATTGTGCAAAGATTCAG	212
36	ndhF-rpl32	SSC	spacer	(A)10	ACAGATATCTATGTTTGGCA	TGCCATGCAACTGATATAGT	200
37	ndhG-ndhI	SSC	spacer	(T)10	ATAGAACAGATATCGAAATGA	AATAGATATGAAACAGAATA	142

### Phylogenetic analysis

**Figure 5:**
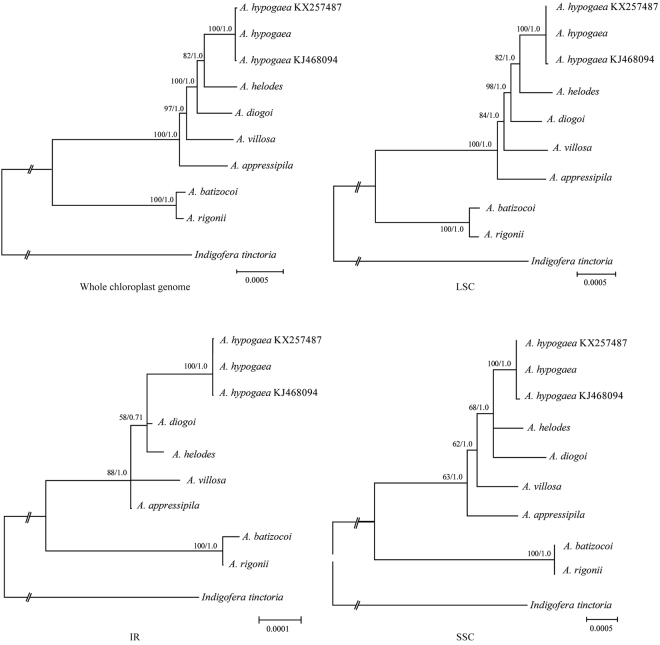
Phylogenetic relationships of the seven *Arachis* species constructed from the complete chloroplast genome sequences using maximum likelihood (ML) and Bayesian inference (BI). ML topology shown with ML bootstrap support value/Bayesian posterior probability given at each node.

We used four datasets (the complete chloroplast genome, the LSC region, the IR region, and the SSC region) to analyze the phylogenetic relationships among members of the genus *Arachis*. The cp genome of *Indigofera tinctoria* was used as the outgroup according to Schwarz, *et al*.^26^. All four datasets produced similar phylogenetic trees with moderate to high support, except for the IR dataset, which had poor support (Fig. 5). The reconstructed phylogeny divided the species into two clades with 100% bootstrap support based on Maximum Likelihood (ML) and Bayesian Inference (BI) analyses. *A. batizocoi* and *A. rigonii* form one clade that is sister to the remaining species with 100% bootstrap support. *A. hypogaea* was closer to *A. helodes* than to *A. diogoi*, *A. villosa*, and *A. appressipila*.

## Discussion

Next generation sequencing (NGS) methods have enabled the rapid and cost-efficient sequencing of plant genomes. In past several years, several studies have reported the development of genetic resources for peanut, including SSRs^14,27^, transcript sequences^28,29^, and draft nuclear genome sequences^1,3^. However, the chloroplast genome is non-recombining and uniparentally inherited, making it a valuable source of information for improving the phylogenetics, species identification, and resolution^18,30,31^. In this study, we generated complete chloroplast genome sequences for seven *Arachis* species using NGS sequencing methods. By comparing the chloroplast genome sequences obtained in this work, we were able to retrieve all genetic resources, including SNPs, microsatellites, indels, and ‘hotspot’ regions.

The seven *Arachis* chloroplast genomes showed a high degree of conservation with respect to genome size and structure, gene number, and also GC content. The genetic divergence was found to be extremely low among the seven *Arachis* chloroplast genomes, as in other angiosperms^32–34^. Within the seven species, only 643 SNPs were detected, which indicated that nucleotide substitution mutations in the chloroplast genome of *Arachis* species are more prevalent than in species of rice^35,36^ and *Panax* ^32^, and less frequent than in species of *Quercus* ^37^. Recently, single nucleotide polymorphisms (SNPs) have become the genetic markers of choice, because they are abundant in genomes and are amenable to high-throughput, automated genotyping assays; consequently, SNPs are increasingly popular in phylogeography, phylogenetics and species identification^38,39^.

In addition to nucleotide substitutions, indels are another important class of genetic variation. The most common indel sizes range from 1 to 200 bp, and mainly occur in non-coding regions^40^. According to our results, the common indels were often less homoplasious than were nucleotide substitutions (Table S2). It has been shown recently that adding microstructural characters significantly increases resolution and support compared to simple substitution-based matrices of chloroplast DNA sequences^41,42^.

SSRs are abundant in the chloroplast genomes of angiosperms, and we identified 101 SSRs in *Arachis*. The most common types are mononucleotide repeats, ranging in size from 10 to 15 nucleotides, mostly A/T. Because chloroplast genome sequences are highly conserved in *Arachis*, chloroplastic microsatellites are transferable across species. Polymorphic SSRs allowed us to develop 37 markers for *Arachis* species (Table 5). These new resources will be potentially useful for population genetic, phylogenetic, and species identification studies in the genus *Arachis*, possibly in combination with the other informative molecular makers from the mitochondrial and nuclear genomes^27,28,43^.

Mutation events in the cp genome are not random, but are clustered in “hotspots,” which result in highly variable regions dispersed throughout the chloroplast genomes^18,44^. We identified eleven variable regions in the *Arachis* chloroplast genome, which enabled the development of novel markers for genetic studies in *Arachis*. The divergent hotspot regions could provide adequate genetic information for phylogenetics and species identification, and can be used to develop novel DNA barcodes for *Arachis*.

The chloroplast genome contains highly informative markers in plant phylogenetics due to its relatively small size, largely uniparental inheritance, conservation of gene number and order. With NGS technology, the chloroplast genome can be obtained efficiently, and much valuable sequence data from the chloroplast genome can be developed for plant relationships^45–48^. These larger datasets can offer opportunities for resolving the most taxa in the plant tree of life, even at the species level^36^.

The genus *Arachis*, including approximately 80 species, were divided into nine sections basing on morphological features, geographic distribution and cytogenetics^4^. Although more molecular makers were explored to evaluate *Arachis* species and sections, the phylogenetic relationships among these them are not fully understood. Here we present a molecular phylogenomics representing nine species. With the chloroplast genome data, they were divided into two groups. One group contains *A. batizocoi* and *A. rigonii*, and the other species, *A. appressipila*, *A. appressipila*, *A. helodes*, *A. villosa*, *A. diogoi*, and *A. hypogaea* were clustered together. These *Arachis* chloroplast genome provided genome-scale information to infer the phylogenetic relationships in *Arachis*.

In this study, we identified indel, SNP, microsatellite, and variable region markers for *Arachis* by comparative analyses of the seven chloroplast genomes. These new resources will be valuable for exploring the variation in *Arachis* populations, as well as for phylogenetics and species barcoding.

## Materials and Methods

### Plant material and DNA extraction

All plant material used in this study was grown in the greenhouse. Fresh leaves of seven *Arachis* species: *Arachis appressipila* Krapov. & W. C. Greg., *Arachis batizocoi* Krapov. & W.C. Greg., *Arachis diogoi* Hoehne, *Arachis helodes* Mart. ex Krapov. & Rigoni, *Arachis hypogaea* L., *Arachis rigonii* Krapov. & W.C. Greg., and *Arachis villosa* Benth. were sampled (Table S1). Fresh leaves from each accession were immediately dried with silica gel prior to DNA extraction. Total genomic DNA was extracted using a modified CTAB method^49^, and the DNA concentration was quantified using a NanoDrop spectrophotometer (Thermo Scientific, Carlsbad, CA, USA). Total DNA samples with concentrations >30 ng μL^−1^ were chosen for Illumina sequencing.

### Chloroplast genome sequencing, assembly and annotation

DNA was sheared to construct a 400 bp (insert size) paired-end library in accordance with the Illumina HiSeq. 4000 standard protocol. The paired-end reads were qualitatively assessed and assembled using SPAdes 3.6.1^50^. Gaps in the cpDNA sequences were filled by PCR amplification and Sanger sequencing. Sanger sequence reads were proofread and assembled with Sequencher 4.10 (http://www.genecodes.com). The four junctions between the inverted repeats (IRs) and the small single copy (SSC)/large single copy (LSC) regions were checked by amplification with specific primers followed by Sanger sequencing^51^. The cpDNA annotation was performed with Plann^52^ using the *A. hypogaea* reference sequence from Genbank (KX257487). The cpDNA genome map was drawn using Genome Vx software^53^.

### Molecular marker development and validation

All sequenced *Arachis* cp genomes were aligned using MIFFT v7^54^, assuming collinear genomes for the full alignment, and then adjusted manually using Se-Al 2.0^55^. Variable and parsimony-informative base sites across the complete cp genomes and the LSC, SSC, and IR regions of the six cp genomes were calculated using MEGA 6.0 software^56^. The p-distances among the *Arachis* chloroplast genomes were calculated with MEGA software to evaluate the divergence among the *Arachis* species.

A sliding window analysis was conducted to calculate the nucleotide diversity (Pi) of the cp genome using DnaSP v5 software. The step size was set to 200 bp, with a 600-bp window length.

For retrieving indel mutations, the multiple sequence alignment was imported into DnaSP v5 software^57^. All indels were initially filtered to separate SSRs (simple sequence repeats) from other indel types.

The cp genome sequences were analyzed to identify potential microsatellites (SSRs) using MISA software (http://pgrc.ipk-gatersleben.de/misa/). The minimum numbers (thresholds) for the SSR motifs were 10, 5, 4, 3, 3, and 3 for mono-, di-, tri-, tetra-, penta-, and hexa-nucleotide repeats, respectively. All of the repeats found were manually verified, and redundant results were removed.

### Phylogenetic analysis

To evaluate the consistency of phylogenetic trees produced from cpDNA regions with different molecular evolutionary rates, we extracted three subsets (LSC, SSC, and IRs) from the complete chloroplast data set, and combined these to produce three types of trees. The lengths of the alignment matrices of these datasets are shown in Table 4. In all the phylogenetic analyses, *Indigofera tinctoria* was used as an outgroup.

The Akaike Information Criterion (AIC) was used in the jModelTest software package v 2.1.3^58^ to compare models of character evolution. Maximum likelihood analysis was performed using the RAxML v 8.0.5 software package^59^ with 1,000 non-parametric bootstrap replicates.

MrBayes 3.2.2^60^ was used to perform a Bayesian inference analysis. The Markov chain Monte Carlo (MCMC) analysis was run for 2 × 5,000,000 generations. Trees were sampled at every 1,000 generations with the first 25% discarded as burn-in. The remaining trees were used to build a 50% majority-rule consensus tree. The analysis was run to completion, and the average standard deviation of split frequencies was <0.01.

## Electronic supplementary material

Supplementary information

Supplementary Dataset 4
