# Author Correction: Development of chloroplast genome resources for peanut (*Arachis hypogaea* L.) and other species of *Arachis*

**DOI:** 10.1038/s41598-021-83717-9

**Published:** 2021-02-11

**Authors:** Dongmei Yin, Yun Wang, Xingguo Zhang, Xingli Ma, Xiaoyan He, Jianhang Zhang

**Affiliations:** College of Agronomy, Henan Agricultural University, Zhengzhou, 450002 China

Correction to: *Scientific Reports* 10.1038/s41598-017-12026-x, published online 14 September 2017

The Data Availability section was omitted in this Article, and is available below.

**Data Availability**

All raw sequencing data generated in this study is available in the NCBI databases under the BioProject accession number PRJNA430760.

