# Supplementary material for: Development of chloroplast genome resources for peanut (Arachis hypogaea L.) and other species of Arachis
Source: Sci Rep. 2017 Sep 14;7:11649. doi: 10.1038/s41598-017-12026-x (PMC5599657; doi:10.1038/s41598-017-12026-x)
Supplement: Supplementary file 1 — Supplementary information [file 41598_2017_12026_MOESM1_ESM.pdf]

Development of chloroplast genome resources for peanut (*Arachis hypogaea* L.) and other species of *Arachis*

Dongmei Yin☂, Yun Wang, Xingguo Zhang, Xingli Ma, Xiaoyan He, Jianhang Zhang

College of Agronomy, Henan Agricultural University, Zhengzhou 450002, China

☂ Correspondence: yindm@126.com, yindm@henau.edu.cn

Supplementary informations

Table S1. Plant material analyzed in this study.

Table S2. Polymorphic indels identified in the analyzed material. Indel events are reported for each of the seven *Arachis* species.

Table S3. Polymorphic SSR loci identified in the analyzed material. For each species, the number of repeats for each SSR is given.

Table S4. SSR primers for each SSR loci.
